# Feasibility Analysis of Machining Cobalt-Chromium Alloy (Stellite-6) Using TiN Coated Binary Inserts

**DOI:** 10.3390/ma15207294

**Published:** 2022-10-18

**Authors:** Saurabh Shah, Anand Joshi, Kamlesh Chauhan, Ankit Oza, Chander Prakash, Raul Duarte Salgueiral Gomes Campilho, Sandeep Kumar

**Affiliations:** 1 Mechanical Engineering Department, L. D. College of Engineering, Gujarat Technological University, Ahmedabad 380015, India; 2 Mechatronics Engineering Department, Parul University, Vadodara 391760, India; 3 Mechanical Engineering Department, Charusat University, Anand 388421, India; 4 Department of Computer Sciences and Engineering, Institute of Advanced Research, Gandhinagar 382426, India; 5 School of Mechanical Engineering, Lovely Professional University, Phagwara 144411, India; 6 Division of Research and Development, Lovely Professional University, Phagwara 144011, India; 7 Departamento de Engenharia Mecânica, Instituto Superior de Engenharia do Porto Rua Dr. Bernardino de Almeida, 4249-015 Porto, Portugal; 8 Division of Research & Innovation, Uttaranchal University, Dehradun 248007, India

**Keywords:** magnetron sputtering, cobalt chromium alloy, titanium nitride, surface roughness, regression

## Abstract

The objective of the study was to check the feasibility of machining Stellite 6, a cobalt–chromium superalloy, using TiN-coated carbide inserts in an end milling operation. The inserts were coated using the magnetron sputtering process. The sputtering power and gas flow rate were considered as the variables during the coating process. The performance of the coated binary carbide insert was checked during the end milling of Stellite 6 (~45 HRC) through an experiment with a Taguchi design. Experimental runs based on an orthogonal array were executed for each insert type to check the feasibility of machining this cobalt-based alloy. Adequate precision and the optimum parametric conditions were determined and are reported in this study. Analysis of variance (ANOVA) with a two-factor interaction model was also undertaken to forecast the key elements influencing surface roughness. Based on the ANOVA model, the depth of the cut, combined with the insert type, was the factor that had the greatest influence on surface roughness, followed by the cutting feed, whereas the cutting velocity had the least significance based on the tests. Moreover, the regression analysis demonstrated that the created model can be used to accurately forecast surface roughness in end milling of Stellite 6 with confidence intervals of 95%.

## 1. Introduction

Cobalt-based compounds are materials that have a cobalt premise alloyed with components, such as chromium, tungsten, nickel, and iron. Alongside nickel-based amalgams, they are utilized in testing conditions that include high temperatures and acids. These alloys are wear-, corrosion-, and heat-resistant; that is, they are sustainable at high temperatures. The predominant use for cobalt-based alloys is in the field of wear-resisting components.

Cobalt-based superalloys are widely employed in applications requiring strong heat, corrosion, and wear resistance [1]. Possessing advantageous characteristics, this material is favoured over others for use in nuclear, aerospace, and sea-water applications [2]. The use of Stellite alloys has emerged in a variety of sectors: oil and gas processing, chemical processing, paper and pulp manufacturing, pharmaceuticals, and medical implant applications. It has been determined that processing alterations, which influence the Stellite alloy’s microstructure, are likely to impede the performance of corrosion [3]. The strengthening of Co-based alloys is generally enhanced by using elements such as tungsten, molybdenum, chromium, and columbium [4,5]. A number of varieties of these alloys are commercially available, which are widely employed nowadays in applications requiring exceptional wear resistance, corrosion resistance, and heat resistance at high temperatures [6]. Stellite alloys are manufactured with the sintering process. Sintered materials are extensively used in the different deposition methods employed for layering on a substrate [7]. The shaping or machining of such materials is normally undertaken with either non-conventional machining processes or grinding processes due to their poor machinability and specific properties. However, both the processes take a long time to complete and, thus, the cost of machining is also high [8]. To overcome this, either turning or milling processes can be used, which require less time to remove the material and, thus, lower the manufacturing cost. Major work has been carried out focusing on the turning process but there is limited work studying milling process. The milling process has a higher material removal rate [9]. Stellite 6 possesses high hardness, and lower thermal conductivity during machining means that it requires high temperature, thus categorizing it as a hard-to-cut material [10]. The difficulty of machining Stellite 6 results in requirements for expensive parts for manufacturing, thus restricting its usage [7]. The difficulty of machining cobalt-based superalloys brings to attention two major concerns: first, work hardening’s effect on tool life and abrasion of the superalloy and, second, the effects on surface stability due to heat generation and plastic deformation in a machined workpiece. With the aim of achieving passable tool life, as well as maintaining the integrity of the machined surface, an analysis of cutting forces, which are a factor in selecting the suitable circumstances and variables for machining, is vital.

Hard-to-cut materials can be machined with a minimum tool–material contact area, ensuring a cutting edge that is sharp and limiting cutting depth. For the lowest heat extraction, a reduced feed rate and cutting speed facilitate machining of such alloys [11]. There are several research studies on end milling that demonstrate that greater feed rates and cutting depths produce greater cutting pressures. Cutting forces have a direct influence by producing a faster cutting rate. Tool selection for the milling process has equal importance when designing machining processes for such superalloys. The tool must have high thermal and wear resistance [12]. The standard tool materials do not work efficiently when machining such superalloys. Specific tool materials or common hard materials with hard coatings (i.e., those produced using chemical vapour deposition (CVD) or physical vapour deposition (PVD)), such as TiN, TiCN, TiAlN, etc., can be used for the machining [13]. Ezugwu et al., 1999 [14], has shown that single-layer TiN-coated inserts produced with PVD provide good surface quality due to the polishing action generated at the cutting edge. Aramcharoen et al., 2008 [15], found that thin-film PVD-coated TiN performed well in resisting flank wear, reduced the chipping, and provided a good surface quality to machine tool steel in micro-milling. PVD-coated tools are well-suited for fine, medium, and rough milling. They are preferred for milling with lower feed rates and/or lower cutting speeds. The PVD coating offers good wear resistance and low friction. The magnetron sputtering method has a good impact on the functionality of the coated parts. This process involves a cathode set as that target that is bombarded by activated ions produced from plasma glow discharge, which is located in front of the target. The act of bombardment results in sputtering (removal) of target atoms, after which they gradually condense on the substrate [16]. Common process parameters considered for the coating are the deposition voltage, substrate temperature, flow rate of gases, sputtering power, sputtering pressure, target–substrate distance, deposition time, etc. Variations in the different coating parameter create substantial effects on the functionality of the surfaces [17,18,19,20]. The sputtering power and the gas flow rate greatly impact the strength and finishing of the coating. As the sputtering power increases, the roughness of the coating increases, and as the gas flow rate increases, the nano hardness of the coating decreases [21]. The evaluating parameters, such as surface roughness, cutting force, tool life, chip morphology, etc., play significant roles in the machining process. Amongst them, the surface roughness has major impacts on the fatigue resistance, lubrication, friction, and wear in assessments of the quality of the machined parts. The surface roughness has a major influence on the cutting parameters. Many researchers have found that the surface roughness is sequentially affected of the cutting feed, cutting speed, and the depth of the cut. Even the use of cutting fluid affects the surface roughness [22,23,24,25,26].

The laser metal deposition of Stellite 6 on 17-4 PH stainless steel was analysed using different process parameters, such as scanning speed and focal length. Increases in the scanning speed could prevent the cracking of the samples during the deposition [27]. The laser-coated cladding on H13 steel was used to enhance the hardness and wear resistance. Stellite 6–Cr_3_C_2_–WS_2_ composite powder was used to cover the H13 steel. It has shown good resistance to friction up to 200 °C, as well as excellent reductions in abrasive wear and adhesive wear due to the self-lubricating phase of the coatings [28]. Stellite 6 parts manufactured by wire arc additive manufacturing (WAAM) exhibit good formation quality. The parts manufactured by WAAM can be made thinner than the cast parts [29].

Aggarwal et al., 2008 [30] examined the power consumption of hard-turning AISI P-20 tool steel (32–36 HRC) with a TiN-coated carbide insert utilising a Taguchi method and an RSM approach. The cutting speed and depth of cut had the highest importance for reducing power consumption after the cryogenic environment. It was discovered that the effects of feed rate and nose radius were negligible. The Taguchi method was shown to be inferior to the RSM technique. Sahoo et al., 2013, utilising a traditional casting procedure, created an Al/SiCp (10% weight) metal matrix composite and investigated its machinability features for turning with a multilayer TiN-coated carbide insert in a dry environment in accordance with Taguchi’s L9 orthogonal array [31]. The regression models, due to their greater R2 values, were very significant. The experimental and anticipated values were similar. Kumar Sahoo and Mohanty, 2013 used Taguchi’s parameter design to optimize parameters for individual responses [32]. A mathematical statistical model was prepared and used to investigate the surface roughness for machining of Stellite 6. The prepared model was also evaluated to determine the technical parameters for longitudinal turning. The mathematical statistical and analytical models allowed precise optimization of the technological parameters [33]. The process of “laser surface alloying” (LSA) is used to maintain and enhance wear characteristics. Refurbishing or improving materials by adding rhenium to the surface layer’s composition could be advantageous, especially for enhancing functional qualities (wear and corrosion resistance). However, given the cost of employing rhenium for this purpose, the addition of rhenium to Stellite 6 has not shown very impressive results [34].

In the present work, the machinability of Stellite 6 material was analysed using an end milling process with coated binary inserts. The inserts were coated with titanium nitride using the magnetron sputtering technique (physical vapour deposition (PVD)) by varying the sputtering power and gas flow rate. Based on process variables including the cutting velocity, cutting feed, and axial depth of the cut, coated inserts were evaluated. The performance of the coated inserts processed in different environments with a range of cutting parameters was evaluated to analyse the surface roughness, and a Taguchi design was utilized for the experiments to optimize the process parameters. A regression model was created and evaluated for suitability.

## 2. Materials and Methods

**Figure 1:**
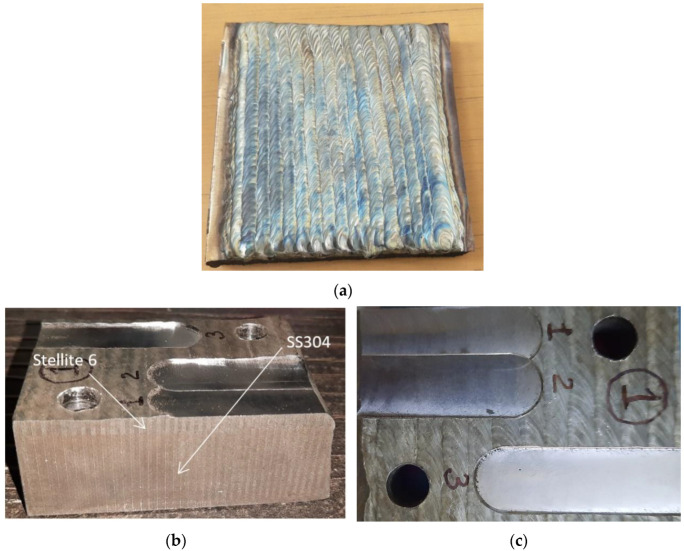
(**a**–**c**) SS304 with Stellite 6 cladding.

For the experimentation, SS304 material of size 150 mm × 150 mm × 25 mm was clad with the cobalt-based super alloy Stellite 6. The cladding thickness of the Stellite 6 was kept to 6 mm and the cladding was undertaken with gas tungsten arc welding (GTAW). Products cladded was Stellite 6 over SS304 are generally beneficial in the oil and gas and petrochemical industries to enhance lifespans by offering resistance to wear. The prepared sample is shown in Figure 1a. Due to the restriction of the dynamometer platform used to mount the workpiece, the sample was cut into six equal pieces with wire cut electro discharge machining (WEDM), as shown in Figure 1b,c.

The chemical composition of the Stellite 6 material is stated in Table 1.

Stellite 6 is a hard (~45 HRC) and difficult-to-cut material. The properties of Stellite 6 material are presented in Table 2.

For all the machining runs of the Stellite 6-cladded sample, a PX10 three-axis CNC milling machine (Jyoti Ltd., Rajkot, India) was used. The machining process was performed under dry run conditions. The levels of parameters were selected using the pilot experiment. For the combination of parameters for the experimentation, an L9 orthogonal array was used for each variety of the coated inserts. The L9 array was prepared using a Taguchi experimental design. The details of the selected process parameters, along with their levels and the associated environment used, are shown in Table 3.

**Figure 2:**
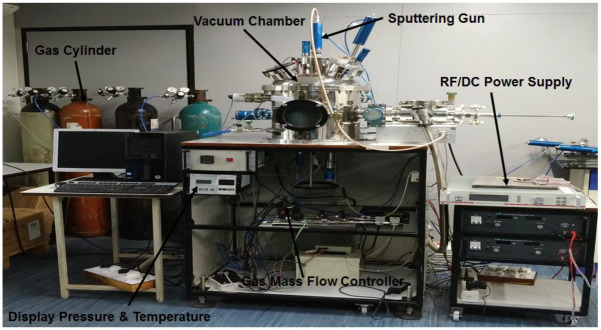
Magnetron sputtering setup.

For machining, tungsten carbide APKT 11 35 (BLOOD model) inserts coated with single layers of TiN (two nos.) (PVD) were used. The magnetron sputtering setup utilized for coating the inserts is shown in Figure 2, and the parameters used in the magnetron sputtering coating are detailed in Table 4.

**Figure 3:**
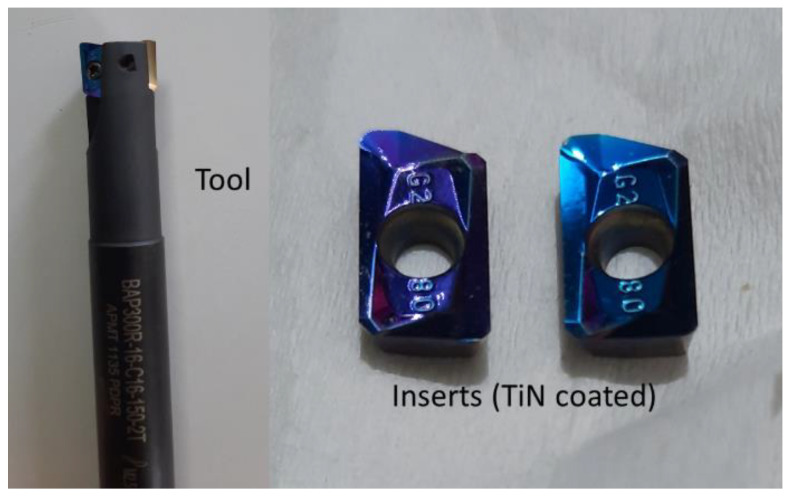
Cutting tool with TiN-coated inserts.

Sputtering powers of 250 W and 300 W and gas flow rates for the nitrogen gas of 8 SCCM and 12 SCCM were used in the PVD for coating. In the present study, tools T1P1 and T1P2 denote the inserts coated at 300 W and 250 W sputtering powers, respectively. Tools T1G1 and T1G2 denote the inserts coated at 12 SCCM and 8 SCCM nitrogen gas flow rates, respectively. A BAP300R-16-C16-150-2T end mill of 16 mm diameter was used. The tool and the coated inserts are shown in Figure 3. For each run, new inserts were used, with a total of 72 inserts used to conduct the total of 36 machining runs. The surface roughness was measured with a Mitutoyo Surf Test SV-2100 surface roughness tester (Mitutoyo, Kawasaki-shi, Japan).

## 3. Results and Discussion

### 3.1. Surface Roughness

Table 5 summarizes the end milling test findings in terms of the surface roughness attained for the Stellite 6 work piece material (~45 HRC) in a dry cutting environment.

**Figure 4:**
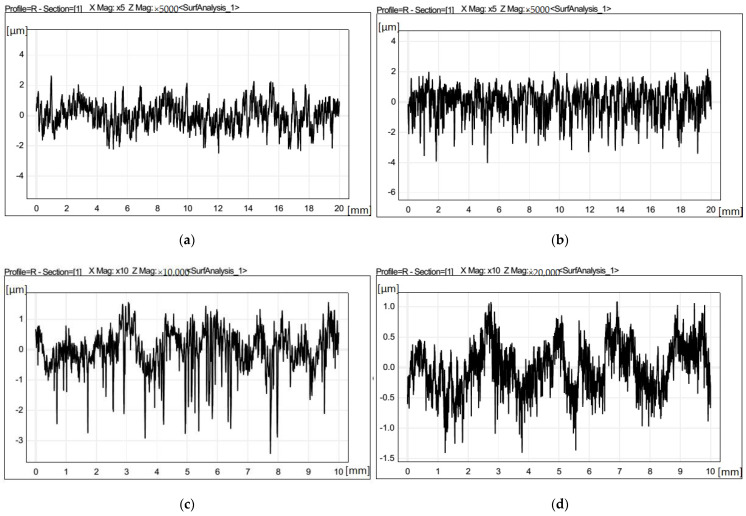
(**a**–**d**) Surface texture measurement results for sample machined with TIP1, TIP2, TIG1, and TIG2.

Figure 4 shows the surface texture results for each of the coated insert configurations.

### 3.2. Analysis of Variance

**Figure 5:**
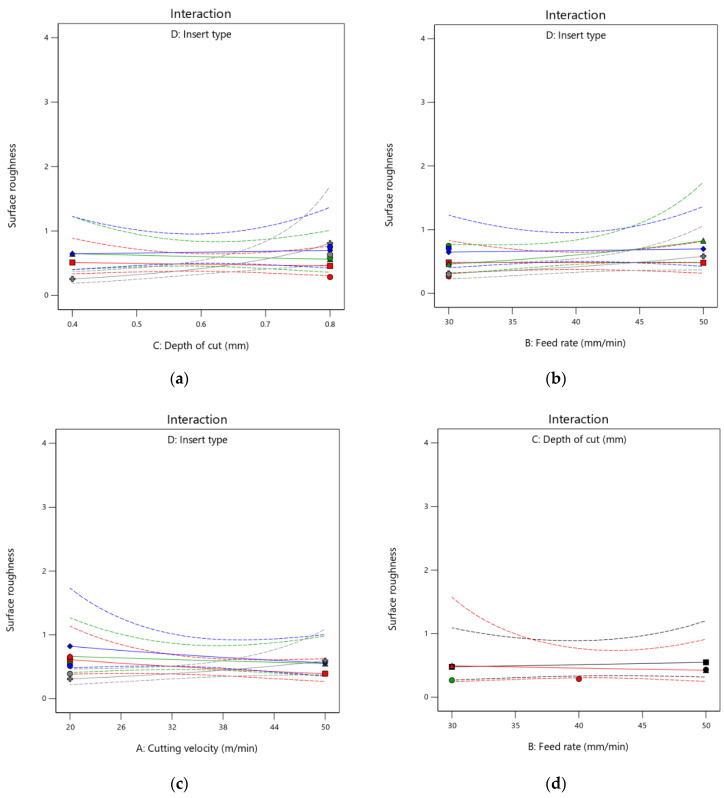

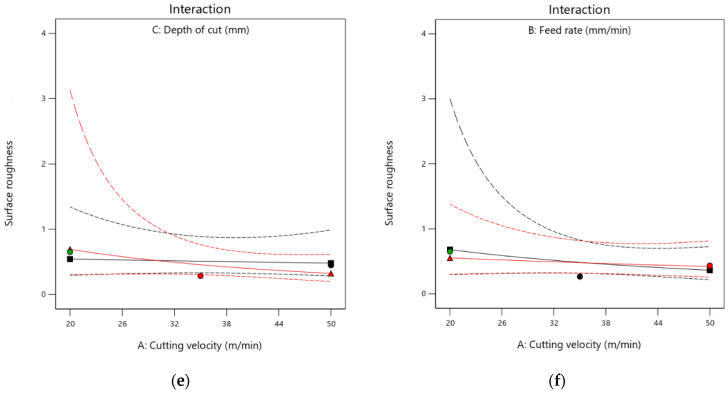
(**a**–**f**) Interaction plots for cutting velocity (A), cutting feed (B), depth of cut (C), and insert type (D) (two-factor interaction).

The goal of the ANOVA was to determine which process variables had a substantial impact on the performance traits. This evaluation highlighted the comparative involvement of machining parameters in governing the performance requirements for the machining response; i.e., the surface roughness during Stellite 6 end milling. This was accomplished by separating the overall range of the adequate precision, which was evaluated by the sum of the squared departures, from the appropriate precision’s overall mean and the contributions of each process parameter and error. Table 6 displays the findings from the variance analysis for the surface roughness. Ninety-five percent confidence intervals were used to determine the outcome of the ANOVA test for the experimental results, and it was found that the *p*-values for the models created for surface roughness were less than 0.05, indicating that the model was significant. From the ANOVA shown in Table 6, it can be concluded that the depth of the cut and the insert type jointly worked as the most significant variables affecting the surface roughness. This interpretation is consistent with the findings of the experiment. Next, the cutting speed and the type of insert were also significant variables, as the *p*-values were less than 0.1. The cutting feed was equally significant for the surface roughness. The cutting velocity and the depth of the cut, as independent factors, were the least significant variables as their *p*-values were greater than 0.1, and they had negligible impacts on the surface roughness of the machining process. Their effects are shown in Figure 5 in the form of interaction plots.

As shown in Figure 5, surface roughness diminished with increasing cutting speed. This was because of the higher force available for the removal of the material. Increases in cutting feed initially reduced surface roughness, but further increases in cutting feed increased the surface roughness. This was as a result of the tool and work piece having less contact time. As the contact time was reduced, the amount of force may not have developed, as can also be observed in Figure 5. This figure also shows that a higher cutting depth increased the cutting temperature. This was due to the greater contact area, which generated more heat through friction, leading to more roughness on the surface.

### 3.3. Statistical Analysis of Surface Roughness

The most important factors— the cutting speed, cutting feed, depth of the cut, and insert type—are notated as A, B, C, and D, respectively. An analytical mathematical model was created for the effective surface finishing criteria during the machining of Stellite 6, with the aid of the test findings for the surface roughness of TiN-coated cutting inserts. The following mathematical model for Ra was created by using multiple linear regression and correlation analysis:

(T1P1): 1/sqrt (Surface Roughness) = 1.85624 + 0.003566 × A − 0.000856 × B − 2.14094 × C − 0.000399 × A × B + 0.037822 × A × C + 0.025277 × B × C(1)

(T1P2): 1/sqrt (Surface Roughness) = 2.65824 − 0.002921 × A − 0.019198 × B − 2.11663 × C − 0.000399 × A × B + 0.037822 × A × C + 0.025277 × B × C(2)

(T1G1): 1/sqrt (Surface Roughness) = 2.03326 + 0.000693 × A − 0.003381 × B − 2.44911 × C − 0.000399 × A × B + 0.037822 × A × C + 0.025277 × B × C(3)

(T1G2): 1/sqrt (Surface Roughness) = 5.19506 − 0.022864 × A − 0.024196 × B − 4.43146 × C − 0.000399 × A × B + 0.037822 × A × C + 0.025277 × B × C(4)

RSM was used to optimize the surface roughness for insert types using cutting parameters. The results for the surface roughness (Ra) from various experimental runs with the four selected factors are shown in Table 5. For each insert type, the Taguchi experiments were repeated 36 times in total. For the statistical analysis of the surface roughness, the 2FI model was selected. ANOVA results for the surface roughness model showed an F-value of 2.39, indicating that the model was statistically significant (*p* < 0.05) at a 95% confidence level (Table 6).

Additionally, the appropriate precision determined for the surface roughness (7.9098) was higher than 4, indicating that the model’s signal was adequate. This model could therefore be used to explore the design space of surface roughness using cutting parameters.

**Figure 6:**
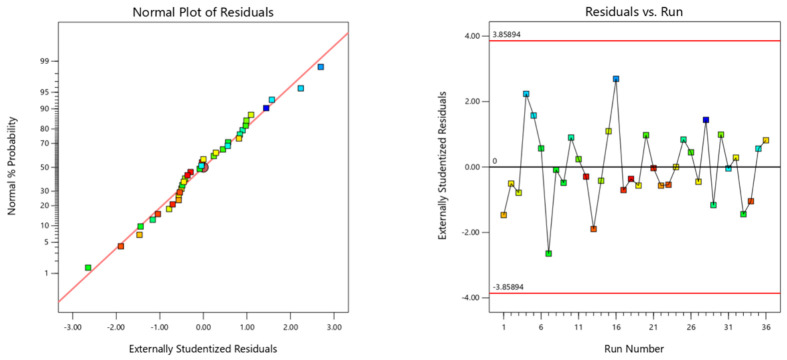
Normal probability and residual vs. run plot.

The normal plots of residuals were produced in Design Expert 13.0.5.0 software to verify that the chosen model was well-matched to the experimental design data. The normal probability plot for the surface roughness, as indicated in Figure 6, indicated a normal distribution for the points in each example following a straight line. In light of the specific models, the data were thus regarded as regularly distributed.

**Figure 7:**
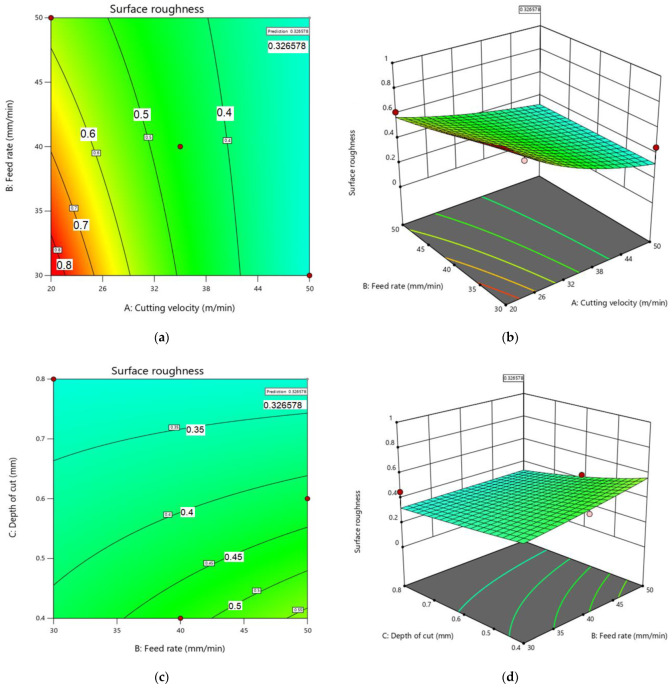

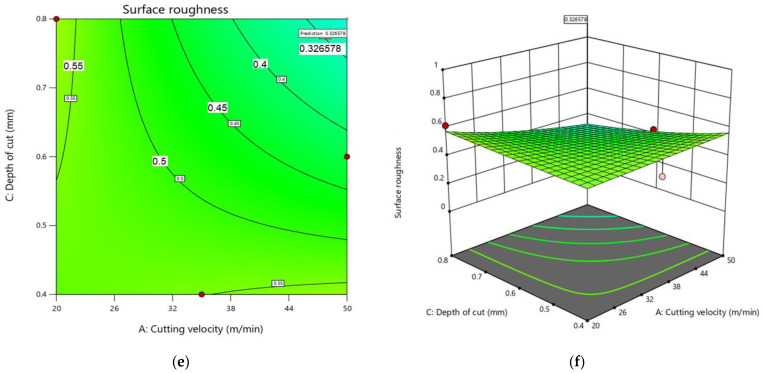
(**a**–**f**) Contour and surface plots for two interactions.

To ascertain the relationship between the response and the independent variables, such as surface roughness, 3D surface and contour plots were plotted by means of RSM (Figure 7a–f). The contour plots specified the interaction of the parameters for the resulting surface roughness. The interaction of the different combinations of parameters and their effects on surface roughness and its trend are shown in the surface plot.

The surface plot for interactive parameters (Figure 7) showed that the optimum position lay inside the experimental region.

**Figure 8:**
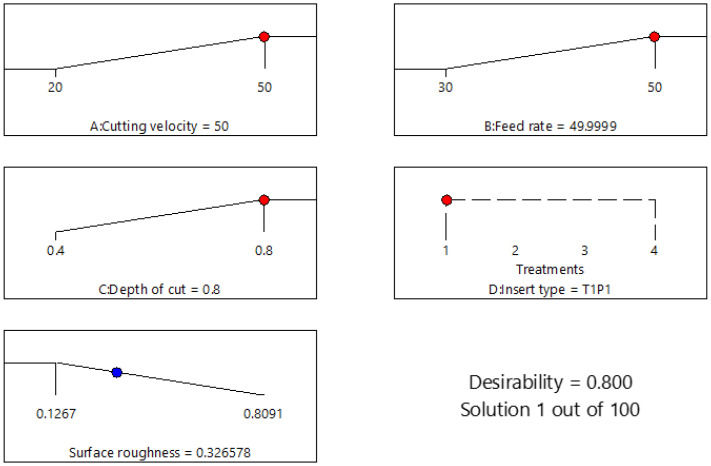
Ramp plot for optimization of surface roughness.

As illustrated in Figure 8, the ramp plot suggested the condition for which the optimum result would be predicted with maximum cutting parameters with minimum surface roughness. The maximized values and optimal selection for the cutting velocity, cutting feed, depth of cut, and insert type were 50 m/min, 50 mm/min, 0.8 mm, and T1P1, respectively. The lowest surface roughness was 0.327, with 0.800 desirability. To validate the statistical model obtained, additional verification experiments were performed at maximized conditions. The average result was 0.306, which was in good agreement with the predicted response.

## 4. Conclusions

The following inferences can be made regarding efficient machining to obtain superior surface quality properties while using hard end milling.

- The TiN-coated tool produced lower surface roughness in the case of machined surfaces and did so within the required range for hard end milling (0.8 μm). This was thought to be caused by the TiN-coated material’s high hardness and wear resistance, low coefficient of friction, and high diffusion barrier qualities;
- The factor that had the greatest impact on surface roughness was the cutting feed. The faster feed rate caused the cutting tool to move across the workpiece too quickly, degrading the surface quality;
- The major experimental work was performed with unconventional machining processes, such as WEDM [36], EDM [37], USM [38], and LBM [37], whereas conventionally it is performed with the turning process [39,40]. In light of the requirements of the industry and the very limited work undertaken with the end milling process, it was chosen for this work. The experimental results showed that, for T1G2, the cutting velocity was 20 m/min, the feed rate was 30 mm/min, and the depth of the cut was 0.4 mm, for which the average surface roughness was 0.1267 µm. The cutting velocity was thought to be the characteristic that had the least impact on surface roughness because the roughness was low at shallower cutting depths with a slower rate;
- It was noted through the analysis of variance (ANOVA) that the depth of the cut and the insert type were the most influential parameters for surface roughness, followed by the cutting feed. The cutting velocity was found to be insignificant in end milling Stellite 6;
- To produce a high surface finish at a faster rate of machining on the Stellite 6 material, a higher cutting speed, higher feed, and higher cutting depth are desired. The best set of parameters for maximal cutting with lower roughness were a cutting velocity of 50 m/min, cutting feed of 50 mm/min, and depth of cut of 0.8 mm, and the best TiN-coated cutting insert was the insert type T1P1, which together produced a surface roughness of 0.327 µm;
- From the collected data, a mathematical model of linear regression for surface roughness was created. In the regression model, the model’s ability to describe the outcomes of all changes was indicated by the R2 value (0.7172), which was close to 1. Thus, the created model can be successfully applied to forecast surface roughness with 95% confidence intervals in the machining of Stellite 6.

## Figures and Tables

**Table 1:** Chemical composition of Stellite 6.

Element	C	Si	Mn	P	S	Cr	Mo	Ni	Fe	W	Co
**Weight (%)**	1.37	1.33	0.24	0.015	0.004	28.63	0.44	2.35	2.64	4.69	Bal.

**Table 2:** Properties of Stellite 6 [35].

Property	Value
Hardness, HRC	~45
Yield Strength, MPa	700
Ultimate Tensile Strength, MPa	896
Elongation, %	<1
Thermal Expansion Coefficient (20–500 °C), µm/m K	11.4–14.2
Density, gm/ cm^3^	8.44
Melting Range, °C	1285–1410
Elastic Modulus, GPa	209

**Table 3:** Machining parameters.

Parameters	Levels			
Cutting Velocity, m/min	20	35	50	
Cutting Feed, mm/min	30	40	50	
Axial Depth of Cut, mm	0.4	0.6	0.8	
Cutting Fluid	Dry			
Down Milling				
Length of Machining, mm	50			
Insert Type	T1P1	T1P2	T1G1	T1G2

**Table 4:** The process parameters for PVD coating.

Substrate	Tungsten Carbide Tip
Target	Titanium
Target Distance, mm	50
Base Pressure of Chamber, Pa	5 × 10^−4^
Sputtering Pressure, Pa	1.5
Substrate Temperature	Unheated
Deposition Time, min.	60
Sputtering Power, W	250, 300
Nitrogen Flow Rate, SCCM	8, 12
Argon Flow Rate, SCCM	12

**Table 5:** Experimental results.

Input Variables					Response Variable
Run No.	Insert Type	Cutting Velocity Vc (m/min)	Feed Rate (mm/min)	Depth of Cut (mm)	Surface Roughness (µm)
1	T1P1	20	30	0.4	0.6879
2	T1P1	20	40	0.6	0.6526
3	T1P1	20	50	0.8	0.6163
4	T1P1	35	30	0.6	0.2661
5	T1P1	35	40	0.8	0.2855
6	T1P1	35	50	0.4	0.4315
7	T1P1	50	30	0.8	0.4526
8	T1P1	50	40	0.4	0.4495
9	T1P1	50	50	0.6	0.4356
10	T1P2	20	30	0.4	0.3562
11	T1P2	20	40	0.6	0.5431
12	T1P2	20	50	0.8	0.7970
13	T1P2	35	30	0.6	0.7469
14	T1P2	35	40	0.8	0.5818
15	T1P2	35	50	0.4	0.6102
16	T1P2	50	30	0.8	0.2269
17	T1P2	50	40	0.4	0.7784
18	T1P2	50	50	0.6	0.8091
19	T1G1	20	30	0.4	0.6415
20	T1G1	20	40	0.6	0.5134
21	T1G1	20	50	0.8	0.7815
22	T1G1	35	30	0.6	0.7047
23	T1G1	35	40	0.8	0.7529
24	T1G1	35	50	0.4	0.6441
25	T1G1	50	30	0.8	0.3667
26	T1G1	50	40	0.4	0.5116
27	T1G1	50	50	0.6	0.6472
28	T1G2	20	30	0.4	0.1267
29	T1G2	20	40	0.6	0.3895
30	T1G2	20	50	0.8	0.5535
31	T1G2	35	30	0.6	0.2984
32	T1G2	35	40	0.8	0.6345
33	T1G2	35	50	0.4	0.444
34	T1G2	50	30	0.8	0.7554
35	T1G2	50	40	0.4	0.3169
36	T1G2	50	50	0.6	0.6654

**Table 6:** ANOVA for the response-surface quadratic model surface roughness for cutting parameters.

Source	Sum of Squares	df	Mean Square	F-Value	*p*-Value
**Model**	3.04	18	0.1691	2.39	0.0391
**A—Cutting Velocity**	0.0031	1	0.0031	0.0435	0.8373
**B—Feed Rate**	0.2500	1	0.2500	3.54	0.0771
**C—Depth of Cut**	0.0490	1	0.0490	0.6934	0.4166
**D—Insert Type**	0.5447	3	0.1816	2.57	0.0882
**AB**	0.0167	1	0.0167	0.2366	0.6329
**AC**	0.0601	1	0.0601	0.8509	0.3692
**AD**	0.5787	3	0.1929	2.73	0.0760
**BC**	0.0119	1	0.0119	0.1689	0.6862
**BD**	0.2394	3	0.0798	1.13	0.3648
**CD**	0.8844	3	0.2948	4.18	0.0219
**Residual**	1.20	17	0.0706		
**Cor Total**	4.24	35			
